# JAK-STAT1/3-induced expression of signal sequence-encoding proopiomelanocortin mRNA in lymphocytes reduces inflammatory pain in rats

**DOI:** 10.1186/1744-8069-8-83

**Published:** 2012-11-13

**Authors:** Melanie Busch-Dienstfertig, Dominika Labuz, Theresa Wolfram, Nicole N Vogel, Christoph Stein

**Affiliations:** 1 Department of Anesthesiology and Critical Care Medicine, Charité Campus Benjamin Franklin, Freie Universität Berlin, Hindenburgdamm 30, 12200, Berlin, Germany; 2 current address: MorphoSys AG, Lena-Christ-Str. 48, 82152, Martinsried/Planegg, Germany

**Keywords:** Pain, Inflammation, Opioid peptides, Immune cells, Neuro-immune interactions, Cytokine signaling.

## Abstract

**Background:**

Proopiomelanocortin (POMC)-derived beta-endorphin_1-31_ from immune cells can inhibit inflammatory pain. Here we investigated cytokine signaling pathways regulating POMC gene expression and beta-endorphin production in lymphocytes to augment such analgesic effects.

**Results:**

Interleukin-4 dose-dependently elevated POMC mRNA expression in naïve lymph node-derived cells in vitro, as determined by real-time PCR. This effect was neutralized by janus kinase (JAK) inhibitors. Transfection of Signal Transducer and Activator of Transcription (STAT) 1/3 but not of STAT6 decoy oligonucleotides abolished interleukin-4 induced POMC gene expression. STAT3 was phosphorylated in in vitro interleukin-4 stimulated lymphocytes and in lymph nodes draining inflamed paws in vivo. Cellular beta-endorphin increased after combined stimulation with interleukin-4 and concanavalin A. Consistently, in vivo reduction of inflammatory pain by passively transferred T cells improved significantly when donor cells were pretreated with interleukin-4 plus concanavalin A. This effect was blocked by naloxone-methiodide.

**Conclusion:**

Interleukin-4 can amplify endogenous opioid peptide expression mediated by JAK-STAT1/3 activation in mitogen-activated lymphocytes. Transfer of these cells leads to inhibition of inflammatory pain via activation of peripheral opioid receptors.

## Background

Inflammatory pain is often refractory to conventional treatments. In addition, currently available opioid analgesics have deleterious side effects such as apnoea or addiction, which have recently lead to an epidemic of overdoses, death and abuse [1]. However, the activation of opioid receptors on peripheral sensory neurons can inhibit pain without central or systemic adverse effects. This can be achieved by exogenous opioids or by endogenous opioid peptides derived from immune cells [2]. These findings are of clinical relevance since human pain is exacerbated by interrupting the interaction between endogenous opioids and their peripheral receptors [3], and is diminished by stimulating opioid secretion [4]. Advantages of targeting endogenous opioids include reduced tolerance, receptor down regulation, desensitization, off-site or paradoxical excitatory effects due to unphysiologically high exogenous agonist concentrations at the receptor [5].

Beta-endorphin_1-31_ is the most prominent opioid peptide eliciting analgesia in peripheral inflamed tissue [2]. In this environment immune cells can locally secrete opioid peptides upon stimulation by stress, corticotropin releasing factor (CRF), catecholamines or chemokines [3,6,7], resulting in the activation of opioid receptors on peripheral terminals of sensory neurons and subsequent analgesic effects [8]. Consistently, stress- and CRF-induced analgesia is reduced in immunosuppressed rats and reconstitution of functional lymphocytes has been shown to reverse this effect [9-11].

In contrast to investigations on the release of opioid peptides from immune cells, the regulation of opioid gene expression and processing in such cells has not been studied in detail so far. Proopiomelanocortin (POMC) is the precursor of beta-endorphin and POMC-related peptides are produced in the pituitary, hypothalamus, immune cells and other tissues [12-14]. The POMC gene comprises three exons that are transcribed into full-length POMC mRNA. Translation of exons 2 and 3 gives rise to a pre-propeptide. In neuroendocrine cells, the formation of the active peptides is accomplished by entering the regulated secretory pathway and involves extensive proteolytic cleavage [15]. Current knowledge about regulatory pathways of beta-endorphin production in lymphocytes is sparse, predominantly because full-length POMC mRNA is difficult to detect in leukocytes [10,16-20]. Using a refined quantitative methodology, we have demonstrated signal sequence-encoding POMC mRNA (exons 2–3) and beta-endorphin in lymph nodes draining inflamed tissue [21]. Others have shown that lymphocytic full-length POMC mRNA can be induced by concanavalin A (ConA), CRF, cytokines or phorbolester in vitro but did not delineate the relevant signaling pathways [22,23]. In pituitary cells, the transcription factors Tpit and Pitx1 [24,25], Nur77, and the janus kinase/signal transducer and activator of transcription (JAK/STAT) are involved in cytokine-induced POMC gene expression [26,27]. The latter pathway is also important in the hypothalamic transcription of the POMC gene induced by leptin [28].

Here we set out to examine cytokines and signaling molecules involved in POMC gene expression in lymphocytes and to test the functional relevance of POMC stimulation for the inhibition of inflammatory pain in vivo. Based on the cytokine expression profile in lymph nodes draining normal and inflamed tissue, in vitro assays were established to test candidate cytokines individually with respect to their potency to elevate POMC mRNA levels in naïve node cells. To enhance cellular activation by mimicking cell-cell contact, lymphocytes were exposed to the mitogen ConA [29]. We hypothesized that peripheral opioid analgesia can be amplified by transfer of cells primed ex vivo to express elevated POMC and beta-endorphin.

## Results

### Exon 2–3 spanning POMC mRNA in lymphocytes is upregulated by IL-4

**Figure 1:**
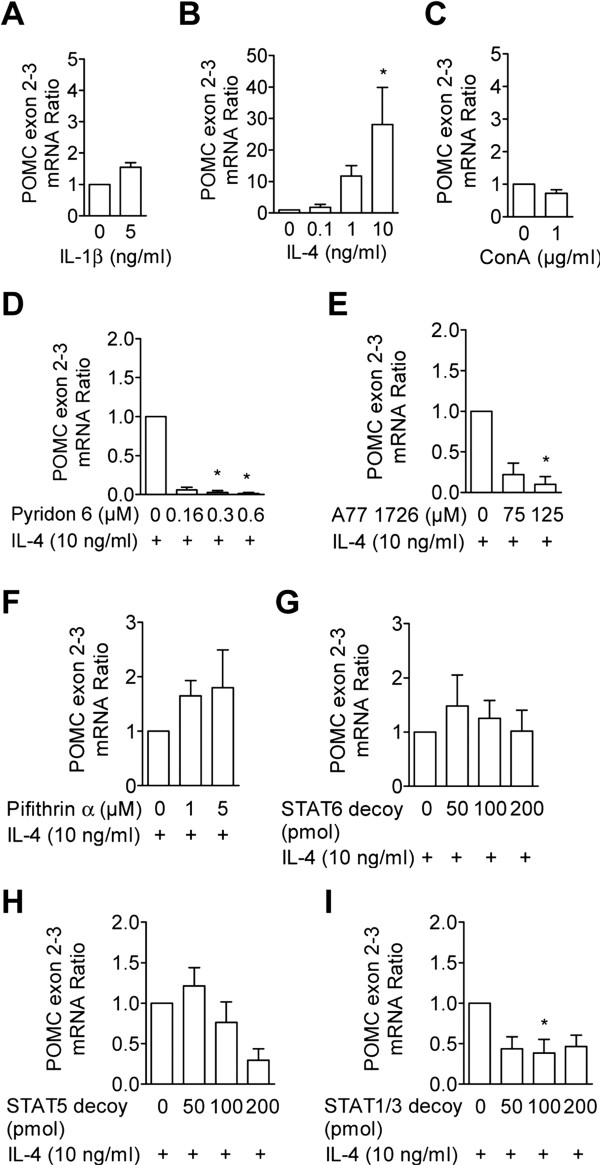
**IL-4 induces POMC exon–3 mRNA expression in lymphocytes via JAK/STAT signaling.** Lymphocytes were incubated for 2 h in the presence of **A**) IL-1β (n = 5), **B**) IL-4 (n = 6), and **C**) ConA (n = 3) at the indicated concentrations. **D**-**F**) Node cells were preincubated with indicated doses of pyridon 6 (n = 5), A77 1726 (n = 4) or cyclic pifithrin-alpha (n = 5) prior to IL-4 addition. **H**-**I**) Lymphocytes were transfected 24 h prior to the addition of IL-4 with STAT6 (n = 5), STAT5 (n = 6), and STAT1/3 (n = 6) decoy oligonucleotides at the indicated concentrations. In **A**-**C** POMC mRNA ratios are given in relation to unstimulated controls; in **D**-**I** POMC mRNA ratios are given in relation to inhibitor-free, IL-4 stimulated controls. Data represent means ± SEM. Statistical analysis was performed on raw data of **A**+**C** using the Wilcoxon signed rank test; raw data of **B** and **D**-**I** were analyzed using the Friedmann and Dunn’s test *P < 0.05; **P < 0.01.

To identify potential regulators of POMC gene expression, we compared the expression profile of inflammatory cytokines in lymph nodes draining normal vs. inflamed paws 2 h following intraplantar (i.pl.) injection of Complete Freund’s Adjuvant (CFA). Among nineteen cytokines analyzed, only IL-1β and IL-4 were significantly up-regulated in comparison to lymph node lysates from healthy animals (Table 1). Stimulation of lymph node-derived naïve lymphocytes with 5 ng/ml interleukin (IL)-1β for 2 h in vitro did not significantly elevate POMC exon 2–3 mRNA transcript levels over unstimulated controls (Figure 1A). Dose-dependent increases of these mRNA transcripts were observed after incubation with IL-4; a significant elevation over control levels was obtained with 10 ng IL-4/ml (Figure 1B). No differences were detectable between untreated and IL-2-, MIP-3α-, MCP-1- (data not shown), or ConA-treated cells (Figure 1C).

**Table 1:** Expression of cytokines in rat lymph node lysates draining normal (untreated) and inflamed (2 h CFA-treated) paws

**Cytokine**	**Untreated**	**Inflamed**
CINC-2	25.33 ± 7.371	29.67 ± 6.0286
CINC-3	13.33 ± 1.155	19.33 ± 6.028
NGFβ	45.67 ± 6.658	51.76 ± 4.509
CNTF	16.67 ± 3.786	18.33 ± 3.055
Fractalkine	26.67 ± 14.01	28.67 ± 0.577
GM-CSF	15.33 ± 9.074	21.00 ± 2.646
IFN-γ	20.33 ± 5.508	23.67 ± 0.577
IL-10	9.00 ± 8.899	15.00 ± 4.583
IL-1α	12.00 ± 8.718	18.67 ± 0.577
IL-1β	12.00 ± 3.000	19.33 ± 2.309*
IL-4	10.50 ± 0.707	14.67 ± 1.155*
IL-6	9.00 ± 4.359	17.00 ± 8.888
Leptin	16.33 ± 11.240	25.00 ± 0.000
LIX	14.67 ± 11.370	24.00 ± 3.464
MCP-1	25.67 ± 6.807	60.00 ± 35.930
MIP-3alpha	13.67 ± 6.807	22.33 ± 10.500
TIMP-1	32.33 ± 11.930	49.67 ± 9.018
TNF-alpha	17.00 ± 1.732	24.67 ± 13.500
VEGF	30.00 ± 5.196	33.00 ± 10.390

*, unpaired *t* test with Welch’s correction (P < 0.05).

Data are expressed as percentages of IgG expression in mean ± SD,

n = 3 animals per group.

### IL-4-induced POMC exon 2–3 mRNA expression in lymphocytes is mediated via JAK and STAT1/3 signaling

**Figure 2:**
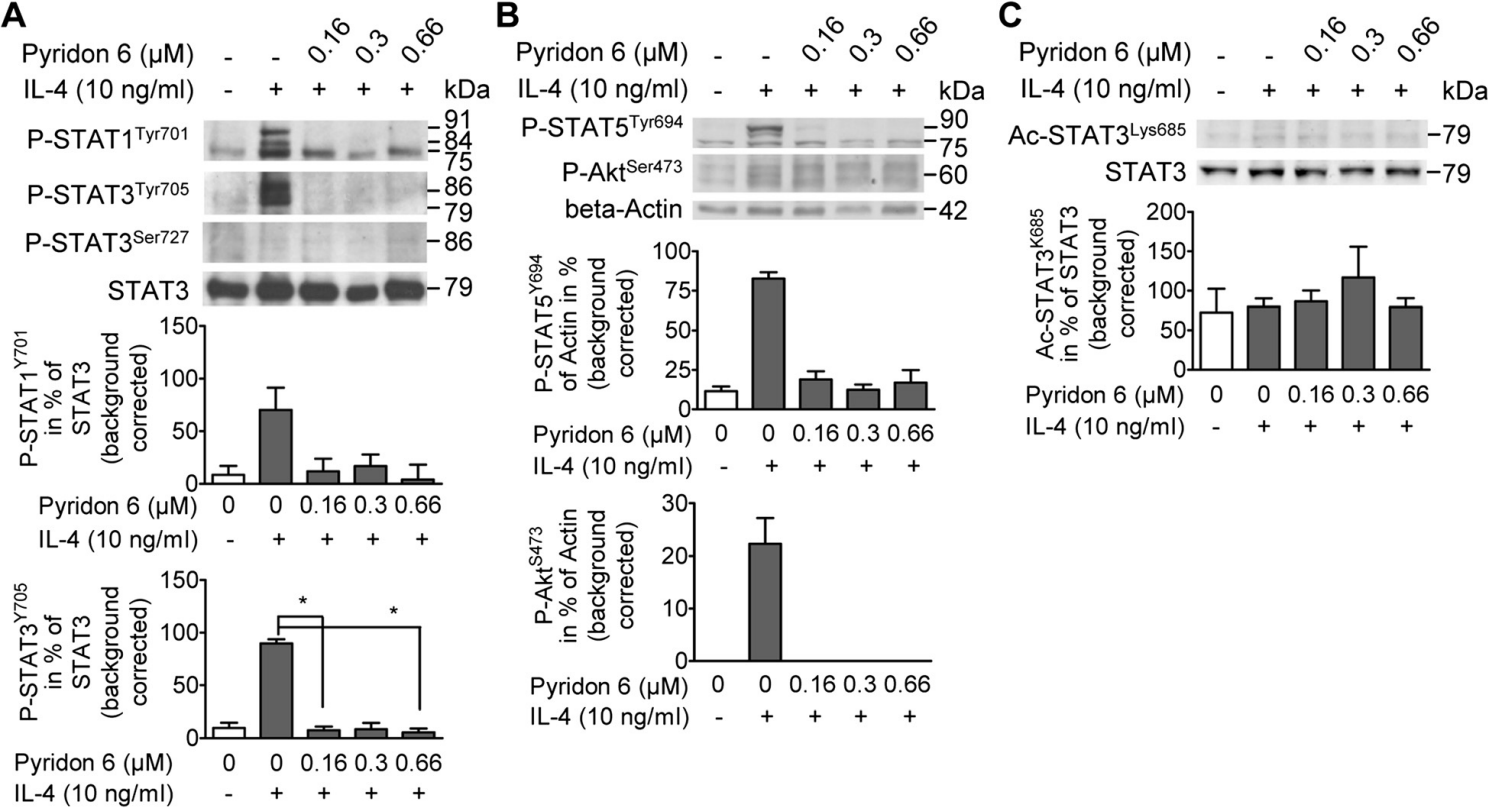
**IL-4-activated JAK signaling results in the phosphorylation of STATs and Akt.** Lymphocytes were preincubated for 30 min prior to the addition of IL-4 with/without pyridon 6. After 2 h IL-4 stimulation, activation of JAK downstream signaling molecules (others than STAT6) was investigated in cell lysates using Western Blotting followed by densitometry. Representative immunoblots are shown; the same blot was probed *sequentially* with different antibodies. Densitometry results are given as mean % expression of loading control ± SEM in the bar graphs; statistical analysis was performed using the Friedman and Dunn’s test; *P < 0.05. **A**) Phosphorylation of STAT1 (Tyrosine 701, 84/91 kDa) and STAT3 (Tyrosine 705, 79/86 kD and Serine 727, 86 kDa) and inhibition by pyridon 6; loading was controlled by staining for non-phosphorylated STAT3 (79/86 kDa). The bar graphs show the mean expression of phosphorylated STAT1 (n = 3 independent experiments), Tyrosine-phosphorylated STAT3 (n = 6 independent experiments, and Serine-phosphorylated STAT3 (n = 3 independent experiments) in % of STAT3, respectively. **B**) Phosphorylation of STAT5 (Tyrosine 694, 90 kDa) and of Akt (Serine 473, 60 kDa) and inhibition by pyridon 6; loading was controlled by staining for beta-actin (42 kDa). The bar graphs show the mean expression of phospho-STAT5 (n = 3 independent experiments) and phospho-Akt (n = 3 independent experiments) in % of beta-actin, respectively. **C**) Acetylation of STAT3 (Lysine 685, 79/86 kDa) and inhibition by pyridon 6; loading was controlled by staining for STAT3 (79/86 kDa). The bar graph shows the expression of Acetyl-STAT3 (n = 3 independent experiments) in % of STAT3. Ac; acetylated; Lys; Lysine; Ser, Serine; Tyr, Tyrosine.

**Figure 3:**
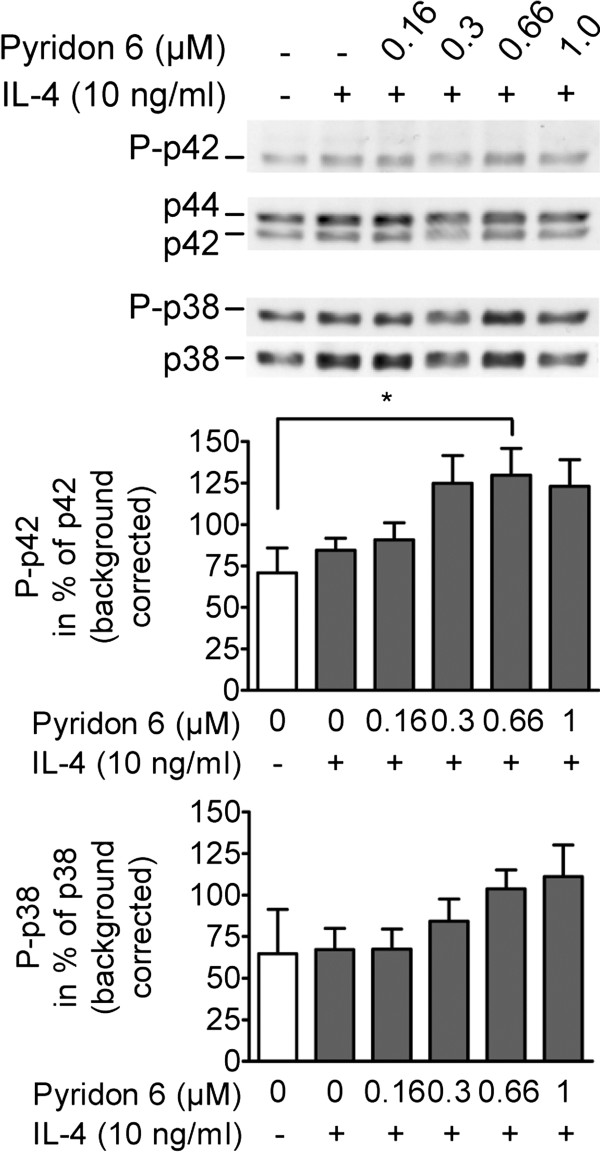
**IL-4 and pyridon 6 do not interfere with MAP kinase pathways.** Lymphocytes were preincubated for 30 min with/without pyridon 6 and stimulated for 2 h with IL-4. Cell lysates were subjected to Western Blotting followed by densitometry. Representative immunoblots show p42 and p38 phosphorylation; loading was controlled by staining for non-phosphorylated p44/42 (42/44 kDa) and non-phosphorylated p38 (38 kDa), respectively. Densitometry results are given as mean % expression of loading control ± SEM in the bar graphs; statistical analysis was performed using the Friedman and Dunn’s test; *P < 0.05. The number of independently performed experiments was n = 4 for p42 and n = 3 for p38 phosphorylation.

The pan-JAK inhibitor pyridon 6 reduced the IL-4-induced elevation of POMC mRNA. This inhibition was significant at concentrations of 0.3 and 0.6 μM (Figure 1D). The JAK1/3 inhibitor A771726 (125 μM), but not the STAT6 inhibitor cyclic pifithrin-alpha, significantly decreased the IL-4-induced elevation of POMC mRNA (Figure 1E, F). IL-4-induced POMC mRNA levels were significantly attenuated by STAT1/3 but not by STAT5 or −6 decoy oligonucleotides (Figure 1G, H, I). After exposure of naïve cells to IL-4, cell lysates were analyzed using Western Blotting. STAT1, STAT3, and STAT5 showed strong Tyrosine-phosphorylations, which were abolished by pyridon 6 pretreatment (see Figures. 2B and C). Significant differences were observed between treatment groups for STAT3 and STAT5 phosphorylation (Friedman test, p < 0.05). Post hoc comparison using Dunn’s test revealed significant differences for Tyrosine-phosphorylation of STAT3 between IL-4 and IL-4 plus 0.16 and 0.66 μM pyridon 6 treated cells. For STAT5 phosphorylation the post hoc comparison remained insignificant. Phosphorylation of STAT3 at Serine 727 was not observed (Figure 2A), while slight STAT3 acetylation at Lysine 685 was observed in unstimulated, IL-4-treated, and pyridon 6 pretreated cells and appeared to be unaffected by the cell treatments (Figure 2C, Friedman test, p > 0.05). Akt phosphorylation at Serine 473 was present after IL-4-stimulation and absent in cells pretreated with pyridon 6 (Figure 2B, Friedman test, p < 0.05). However, post hoc comparison for Akt phosphorylation remained insignificant. Phosphorylation of extracellular-signal regulated kinase (ERK) 2 (p42) and of mitogen-activated protein kinase p38 remained largely unaffected by IL-4-stimulation (Figure 3) but phosphorylation of both kinases tended to increase in IL-4 plus pyridon 6 treated cells. Significant differences between treatment groups were observed for p42-phosphorylation (Friedman test, p < 0.05). Post hoc comparison using Dunn’s test revealed significant differences for p42-phosphorylation between untreated controls and IL-4 plus 0.66 μM pyridon 6 treated cells.

### IL-4 treatment elevates beta-endorphin content and release from mitogen-activated lymphocytes

**Figure 4:**
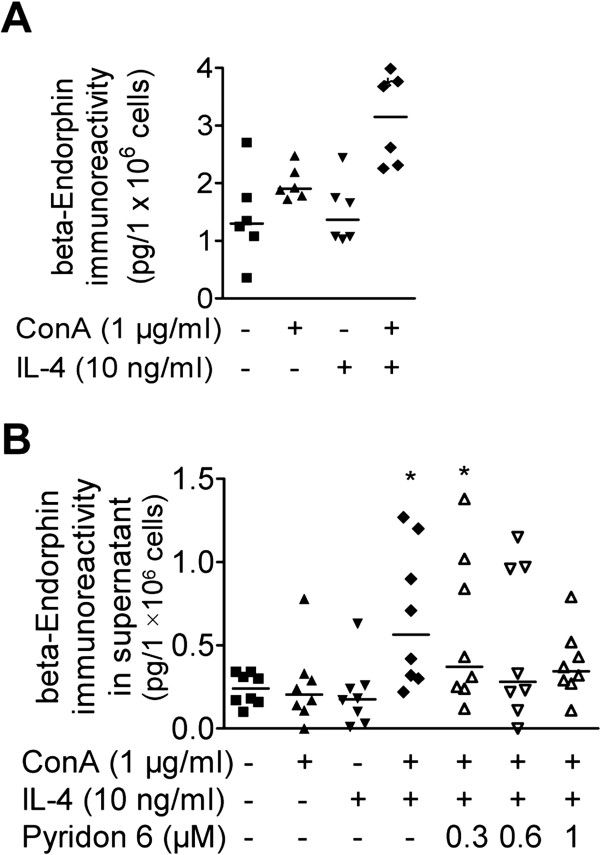
**Cellular levels of beta-endorphin immunoreactivity increase in ConA-activated and IL-4-stimulated lymphocytes.A**) 24 h after cell stimulation with/without IL-4 (10 ng/ml) and ConA (1 μg/ml), beta-endorphin immunoreactivity was determined in cell lysates. Data represent the beta-endorphin immunoreactivity in pg/1.0E+06 cells of n = 6 independent experiments; median is indicated by the horizontal line. **B**) Ionomycin-induced release of immunoreactive beta-endorphin was determined using EIA in the supernatant of cells stimulated for 24 h with/without IL-4 (10 ng/ml), ConA (1 μg/ml), and pretreated with pyridon 6 at the indicated doses. Data represent the beta-endorphin immunoreactivities in pg/1.0E+06 cells of n = 8 independent experiments; median is indicated by the horizontal line. All data were analyzed using Friedmann and Dunn’s Test in comparison to unstimulated controls (*P < 0.05).

Cellular amounts of immunoreactive beta-endorphin did not change in naïve lymphocytes stimulated with IL-4 for 24 h (Figure 4A). To mimick cell activation, naïve lymphocytes were incubated for 24 h with ConA, which had no effect on the cellular levels of immunoreactive beta-endorphin (Figure 4A). However, the combined stimulation with ConA and IL-4 significantly increased contents of immunoreactive beta-endorphin (Figure 4A). Vesicular release was induced by ionomycin-treatment. Extracellular levels of immunoreactive beta-endorphin were significantly higher than controls when cells were pre-stimulated with combined but not separate ConA and IL-4 (Figure 4B). Ionomycin-induced release of immunoreactive beta-endorphin from ConA/IL-4 stimulated cells was not significantly influenced by up to 1 μM pyridon 6 pretreatment (Figure 4B).

### Transfer of mitogen-activated lymphocytes pretreated with IL-4 restores opioid antinociception in immune cell-depleted rats

**Figure 5:**
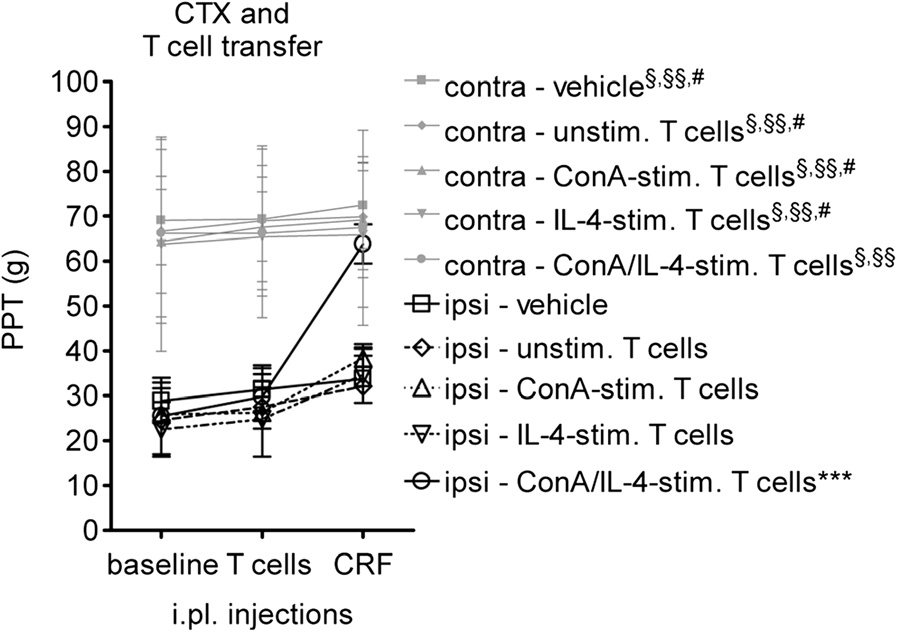
**Transfer of cytokine-stimulated T cells restores CRF-induced antinociception in immunosuppressed rats with hindpaw inflammation.** Cyclophosphamide (CTX)-pretreated rats with CFA-induced hindpaw inflammation were injected i.pl. with untreated, ConA-, IL-4- or ConA/IL-4-pretreated T cells. PPT were determined in the ipsilateral and contralateral paw at baseline, after T cell transfer, and after i.pl. CRF injection. Controls received i.pl. vehicle. Data represent means ± SD (n = 7–8 animals per group). PPT values were analyzed for effects of the injection (baseline, T cell transfer, and CRF-injection) and between paws (ipsi versus contralateral) using Two-Way RM ANOVA and Bonferroni’s Test. §, Ipsilateral PPT are significantly lower than contralateral values at baseline (P < 0.001); §§, Ipsilateral PPT are significantly lower than contralateral values after T cell-injection (P < 0.001); #, Ipsilateral PPT are significantly lower than contralateral values after CRF-injection (P < 0.001). Effects of treatment (vehicle versus unstimulated, ConA-, IL-4-, and ConA/IL-4-stimulated T cells) and injection (baseline, T cell transfer, and CRF-injection) on ipsilateral PPT values were analyzed using Two-Way RM ANOVA and Bonferroni’s Test. ***, Ipsilateral PPT are significantly elevated in recipients of ConA/IL-4-stimulated T cells after CRF-injection (P < 0.001).

**Figure 6:**
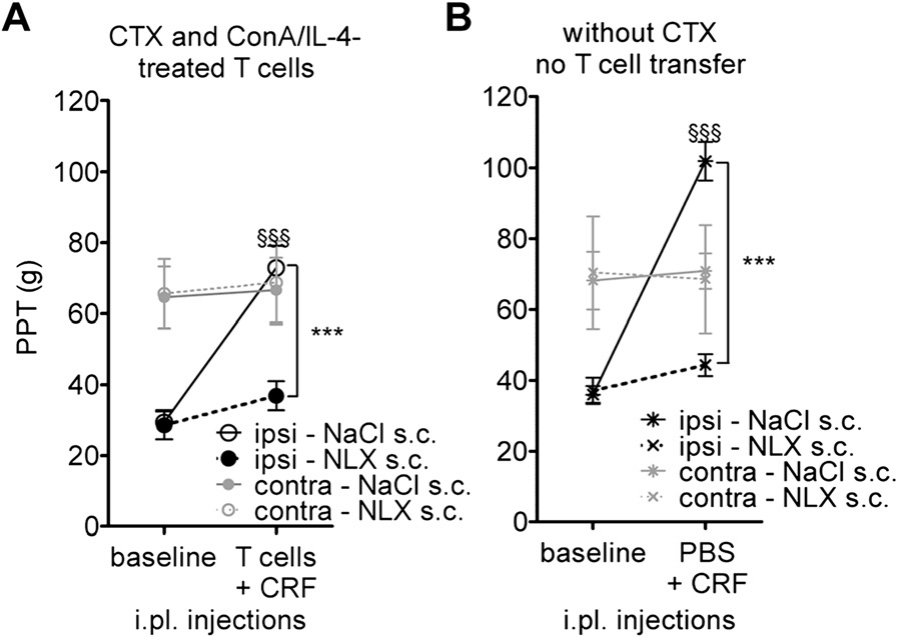
**CRF-induced antinociception in recipients of activated, ConA/IL-4-stimulated T cells is opioid mediated.A**) NLX reversibility of CRF-induced antinociception in CTX-treated recipients of T cells stimulated with ConA/IL-4. PPT were determined at baseline and after i.pl. injections of T cells and CRF. Controls received s.c. NaCl. **B**) CRF-induced antinociception and NLX-reversibility in non-immunosuppressed rats. PPT were determined at baseline and after CRF injection. Data represent means ± SD (n = 7–8 animals per group). Effects of the injection (baseline versus CRF) and of the treatment (saline versus NLX) on ipsilateral PPT were analyzed using Two-Way RM ANOVA and Bonferroni’s Test. §§§, CRF injection significantly elevated PPT in NaCl-treated animals (P < 0.001); ***, CRF-induced elevation of PPT is significantly reduced by NLX (P < 0.001).

Four days after i.pl. injection of Complete Freund’s Adjuvant (CFA), paw pressure thresholds (PPT) in inflamed (ipsilateral) paws were significantly lower than in noninflamed (contralateral) paws of rats immunosuppressed by cyclophosphamide (CTX, Figure 5). Intraplantar transfer of unstimulated or stimulated cells did not change the reduced PPT (hyperalgesia) in inflamed paws in comparison to the baseline levels (Figure 5). However, i.pl. injection of 1.5 ng CRF completely reversed hyperalgesia in paws injected with ConA/IL-4-stimulated T cells compared to all other groups, such that PPT were similar to contralateral noninflamed paws (Figure 5). CRF-induced increases of ipsilateral PPT values were significantly higher in animals receiving 1×10^5^ (63.8 ± 4.4 g) and 5×10^5^ (65.0 ± 7.3 g) ConA/IL-4 treated cells in comparison to 10×10^5^ cells (53.8 ± 8.0 g) (One-Way ANOVA and Bonferroni’s Test, P < 0.05). Therefore, subsequent experiments were performed with the lowest cell number. Four days after i.pl. CFA, PPT were analyzed in immunosuppressed (Figure 6A) and in immunocompetent (Figure 6B) animals pretreated with s.c. NLX prior to CRF injection. PPT in immunosuppressed versus immunocompetent rats were slightly but significantly lower in both contralateral (64.4 ± 1.0 g versus 68.1 ± 1.0 g, respectively; unpaired *t*-test, P < 0.05, 8 rats per group) and in inflamed paws (29.4 ± 1.2 g versus 36.04 ± 0.8 g, respectively, unpaired *t*-test, P < 0.05, 8 rats per group). Baseline PPT in inflamed paws were not influenced by NLX. In recipients of ConA/IL-4-stimulated cells, NLX completely reversed CRF-induced increases of PPT (Figure 6A). In immunocompetent rats, CRF-induced PPT elevations in inflamed paws were significantly higher than in contralateral paws. This effect was abolished by NLX (Figure 6B).

### STAT3 and Akt are phosphorylated in lymph nodes draining inflamed tissue *in vivo*

**Figure 7:**
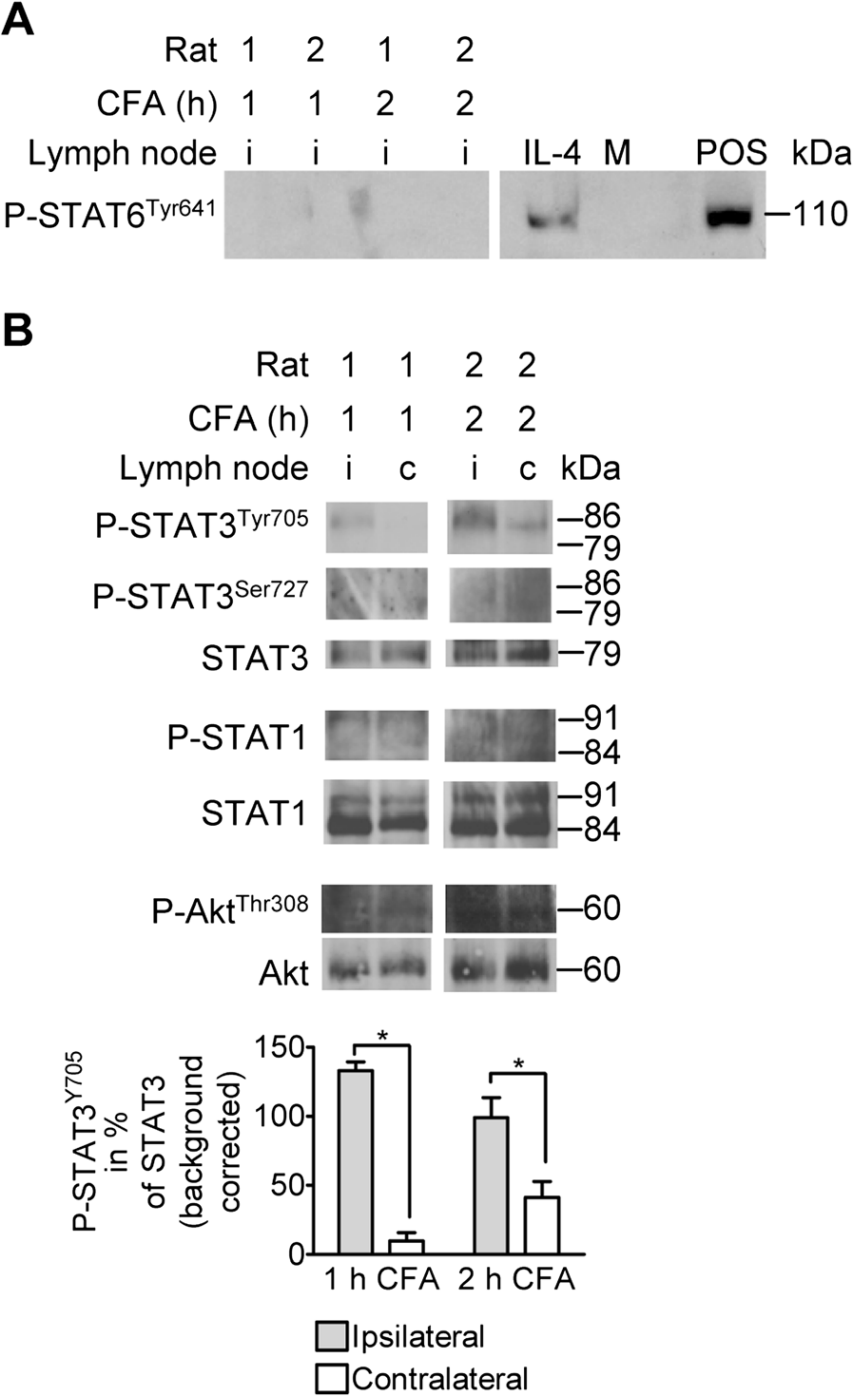
**Phosphorylation of JAK downstream targets in cells from lymph nodes draining inflamed and noninflamed paws.** Popliteal lymph nodes (i, ipsilateral; c, contralateral) were dissected from two animals each at 1 and 2 h post i.pl. CFA injection: Cells were isolated and cell lysates were analyzed using Western Blotting. Densitometry results are given as mean % expression of loading control ± SEM in the bar graphs. **A**) Tyrosine 641-phosphorylation of STAT6 (110 kDa) in CFA- and IL-4-stimulated lymphocytes, two out of 6 nodes analyzed per time point are shown. A commercially available phospho-STAT6 positive control (POS, BioLabs) was run on the same gel. M = protein marker. **B**) Tyrosine 705 (79/86 kDa)- and Serine 727 (86 kDa)-phosphorylation of STAT3, non-phosphorylated STAT3 (79 kDa), phosphorylated STAT1 (84/91 kDa), non-phosphorylated STAT1 (84/91 kDa), Threonine 308-phosphorylated Akt (60 kDa), and non-phosphorylated Akt (60 kDa). Stainings were performed on 3 nodes per time point; the staining for Tyrosine-phosphorylated STAT3 was performed on 6 nodes per time point. Statistical comparison of the ipsi- and contralateral side was performed using the Wilcoxon Matched Pair test; *P < 0.05. Representative immunoblots are shown. The same blot was probed *sequentially* with different antibodies. Lanes shown were run on the same gel, noncontiguous lanes are separated by thin white lines. Ser, Serine; Thr, Threonine; Tyr, Tyrosine.

At 1 and 2 h after induction of CFA-inflammation in vivo, phosphorylated STAT6 was undetectable in cells isolated from ipsi- and contralateral popliteal lymph nodes (Figure 7A). Tyrosine-phosphorylation of STAT3 was observed in cells from ipsi- and contralateral nodes (Figure 7B). Densitometry showed that at both 1 and 2 h post CFA-injection, this phosphorylation was significantly stronger in the ispilateral than in the contralateral node cells (Wilcoxon signed rank test, P < 0.05). Serine-phosphorylation of STAT3 and Tyrosine-phosphorylation of STAT1 were not detectable in cells from ipsi- or contralateral nodes (Figure 7B). Despite background, Threonine-phosphorylation of Akt was detectable in cells from the ipsi- and contralateral nodes, but no reliable densitometric analysis could be performed.

## Discussion

In the present study we found that: i) signal sequence-encoding POMC mRNA expression can be induced in naïve lymph node-derived cells by IL-4 stimulation in vitro, ii) POMC exon 2–3 mRNA up regulation by IL-4 is at least partially mediated via the JAK-STAT pathway involving Tyrosine-phosphorylated STATs 1 and −3, but not STAT5, STAT6, ERK 2 or p38 MAPK, iii) IL-4 induces beta-endorphin production in mitogen-activated lymphocytes, and iv) in vivo transfer of IL-4-stimulated, mitogen-activated T lymphocytes restores CRF-induced, opioid-mediated analgesia in immune cell-depleted animals.

Our previous studies have shown that the levels of signal sequence-encoding POMC mRNA in the draining lymph node increase as early as 2 h post induction of paw inflammation in rats [21]. In neuroendocrine cells the translational product of such mRNA transcripts - in contrast to that of truncated POMC transcripts lacking exon 2 - can enter the secretory pathway [30], which is a prerequisite for the formation and secretion of biologically active POMC-derived peptides including beta-endorphin. However, the regulation of POMC gene expression and processing in immune cells has not been studied in detail so far. We now identified elements that are involved in the transcriptional regulation of POMC expression in lymphocytes. ConA did not enhance POMC exon 2–3 mRNA levels within the relative short time frame of 2 h. Others found ConA-induced POMC mRNA elevation in splenocytes incubated for 21 h with this mitogen [22]. In our experiments IL-1β treatment only slightly elevated POMC exon 2–3 mRNA levels after 2 h. This resembles previous findings in human dermal endothelial and in corticotroph AtT-20 cells [31,32]. Similarly, IL-2 did not elevate the POMC mRNA in our study or in AtT-20 cells [32,33] but IL-4 induced a considerable increase. So far this cytokine was not investigated with respect to POMC mRNA expression in lymphocytes but it was found to stimulate proenkephalin mRNA in peripheral blood mononuclear cells [34].

The predominant pathway of IL-4-induced gene transcription in T and B cells involves JAK1/3 and STAT6 activation [35]. This was also found in case of the μ-opioid receptor [36]. Thus, we hypothesized that the induction of lymphocytic POMC gene expression by IL-4 is mediated via the JAK-STAT6 pathway. Indeed, the IL-4 effect was fully blocked by the pan JAK inhibitor pyridon 6 and by the JAK1/3 inhibitor A771726, but not by the proposed STAT6 inhibitor cyclic pifithrin-alpha. In addition, it was not attenuated by STAT6 but partially by STAT1/3 decoy oligonucleotides. Others demonstrated that phospho-STAT3 activated the POMC promoter through an indirect mechanism requiring an SP1-binding site [28], and that STAT3 and the AP-1 protein complex can cooperate in driving transcription [37]. In pituitary corticotrophs leukemia inhibitory factor induced POMC gene expression via binding of phosphorylated STAT1 and −3 homo- and heterodimers to the promoter [38]. In line with these findings, we detected considerable STAT1 phosphorylation in IL-4 treated lymphocytes. Also, following IL-4 stimulation STAT3 was strongly phosphorylated at Tyrosine 705, which was blocked by the pan JAK inhibitor pyridon 6. This is in agreement with IL-4-induced, JAK3-mediated phosphorylation of STAT3 in naïve cytotoxic T cells [39]. We did not observe STAT3 phosphorylation at Serine 727. Others demonstrated that DNA binding of STAT1 or −3 is not affected by Serine-phosphorylation [40], indicating that Tyrosine-phosphorylation is sufficient for the induction of gene transcription. In line with this notion, leukemia inhibitory factor-induced POMC transcription in the pituitary was abrogated by mutated STAT3 containing Phenylalanine instead of Tyrosine 705 [41].

IL-4 can also activate the phosphoinositide 3-kinase kinase/protein kinase B (Akt) pathway [42]. Concordantly, we found Akt phosphorylation. In addition, this pathway is known to activate Ras-Raf-Mitogen-Activated Protein kinases (MAPK) including phospho-p44/42 (ERK 1 and 2). We found that ERK 2 was already highly phosphorylated in unstimulated lymphocytes and was apparently not changed after treatment with IL-4 or with pyridon 6. Together, these findings suggest that the MAPK pathway is not essential for IL-4-induced POMC gene expression in lymphocytes, which resembles findings in AtT-20 cells [41]. This is also supported by the lack of STAT3 Serine-phosphorylation after IL-4 treatment, which would be expected via the Ras-Raf-MAPK pathway activation [43]. In contrast to human neutrophils [44], we found that p38 MAPK was phosphorylated in both naïve and IL-4-stimulated lymph node-derived cells. Additionally, phosphorylation of p38 was not significantly affected by pyridon 6. Thus, we conclude that p38 is not involved in IL-4-induced POMC gene expression in lymphocytes.

We then analyzed whether IL-4-treatment increased the cellular beta-endorphin content and in vivo antinociceptive function. To obtain significant opioid peptide levels and release in vitro, we had to prime naïve cells with the mitogen ConA, similar to others [11]. This suggests that POMC gene expression and precursor processing are independently regulated in lymphocytes. Inflammatory cells express POMC processing enzymes [45,46], but their functional role in the regulation of processing pathways and beta-endorphin production have not been elucidated. To investigate antinociceptive effects of T cell-derived opioids, we used immune cell-depleted rats and stimulation with CRF, which has been shown to release opioid peptides in vitro and in vivo [9,16]. In recipients of ConA/IL-4-stimulated T cells this resulted in strong antinociception in inflamed paws. In contrast to findings after intravenous cell transfer [11], this effect did not increase with rising cell numbers. Since we administered the cells directly to the site of inflammation, lower numbers may be required. The same CRF dose injected into immunocompetent animals induced a stronger antinociceptive effect than in immunosuppressed T cell recipients, indicating that CRF was not the limiting factor. The antinociceptive effect was blocked by naloxone-methiodide, consistent with the notion that it was mediated by opioid receptors on peripheral terminals of sensory neurons [8]. Together with our in vitro data, these findings indicate that the production of biologically active beta-endorphin is enhanced by treatment of mitogen-activated lymphocytes with IL-4, and that this strategy can be used to amplify opioid inhibition of inflammatory pain in vivo. Thus, we have discovered a new mechanism, adding to previous reports showing antinociceptive effects of IL-4 via the inhibition of pro-inflammatory cytokines [47,48]. In those studies, pain thresholds were determined 30 min after IL-4 injection and the effects were not reversed by naloxone. In our experiments, the opioid-dependent antinociceptive effects produced by passively transferred T lymphocytes pretreated for 24 h with IL-4 plus ConA were detected only after injection of CRF. This further supports the concept that IL-4 induces the production rather than the release of opioid peptides in activated lymphocytes.

Finally, to obtain information on the JAK/STAT signaling molecules at early stages of inflammation in vivo, we analyzed lymph nodes dissected after induction of unilateral paw inflammation by CFA. Phosphorylated STAT6 was undetectable in all tissues. This finding indicates that IL-4 was not released after CFA-inoculation, at least not at substantial concentrations. However, STAT3 Tyrosine-phosphorylation was increased in cells from ipsilateral lymph nodes. Taken together, these findings indicate that the in vivo elevation of POMC mRNA may be due to STAT3 phosphorylation and this effect can be mimicked in vitro by stimulating naïve lymphocytes with IL-4.

In summary, our present and previous data show that beta-endorphin-containing lymphocytes infiltrating inflamed tissue can produce opioid mediated antinociception [11,49,50]. This is also supported by the present finding of increased nociception in immunosuppressed as compared to immunocompetent animals with hind paw inflammation. Furthermore, we have now demonstrated that POMC and beta-endorphin production as well as pain inhibition can be significantly augmented by IL-4-induced activation of the JAK/STAT pathway. This should spawn innovative approaches to pain therapy, for example antigenic stimulation (similar to vaccination), clonal expansion, or genetic manipulation of such cells [51]. Pain relief via enhancement of endogenous opioid production in immune cells may overcome limitations of conventional analgesics such as addiction, paradoxical hyperalgesia, cognitive impairment, nausea and constipation induced by opioid drugs, or gastrointestinal ulcers, bleeding, and cardiovascular complications produced by cyclooxygenase inhibitors.

## Conclusion

The expression of POMC and beta-endorphin in lymphocytes is apparently linked to anti-inflammatory cytokines and JAK-STAT1/3 activation. Interleukin-4 effectively stimulated POMC transcription in naïve cells. Evidently, precursor processing is regulated independently from gene transcription by so far unidentified factors during cell activation. Our data provide a novel in vitro model to study the molecular mechanisms involved in opioid peptide synthesis in such cells and outline novel approaches to pain treatment by promoting production of immune cell derived opioid peptides.

## Materials and methods

### Experimental animals and induction of inflammation

Experiments were approved by the animal care committee of the State of Berlin and strictly followed the guidelines of the International Association for the Study of Pain [52]. Male Wistar rats (225–300 g, Charles River Breeding Laboratories) received an i.pl. injection of 0.15 ml Complete Freunds’ Adjuvant (CFA, Calbiochem, La Jolla, CA, USA) into the right hind paw under brief isoflurane (Rhodia Organic Fine Ldt., Bristol, UK) anesthesia. The inflammation remained confined to that paw throughout the observation period.

### Cytokine array

Popliteal lymph nodes draining normal and inflamed hind paws (2 h post CFA) were dissected, homogenized, and lyzed. Expression of cytokines was analyzed using RayBio^TM^ Rat Cytokine Antibody Array 1.1 kits (RayBiotech, Inc., Norcross, GA, USA) following the manufacturer’s instructions. Array-membranes carrying antibodies for the detection of nineteen cytokines and anti-rat IgG (loading control) were incubated with the lymph node lysates (500 μg protein/membrane) for 2 h. After washing, the membranes were incubated for 2 h with a mixture of biotin-conjugated antibodies, followed by peroxidase-conjugated streptavidin. Immunoreactive dots were subsequently visualized using an enhanced chemiluminescence (ECL) system, and membranes were exposed to autoradiograph hyperfilms for 10 to 30 seconds.

### Preparation of lymphocytes

Healthy rats were sacrificed by isoflurane overdose. Popliteal, axillary, and inguinal lymph nodes were dissected and pooled. Our previous flow cytometry analyses had shown that about 95% of cells residing in naïve nodes express the hematopoietic cell marker CD45, 70-80% are CD3^+^ T lymphocytes, and 20-25% are IgG kappa light chain^+^ B lymphocytes [21]. Lymphocytes were dissociated from surrounding tissue using 40 μm mesh cell strainers and were cultured in RPMI-1640 medium containing 1% penicillin/streptomycin under standard culturing conditions (37°C and 5% CO_2_) unless otherwise stated.

### Cytokine stimulation and inhibitor treatment of lymphocytes

Naïve lymphocytes were stimulated for 2 or 24 h with 0.1 – 10 ng/ml of rat recombinant cytokines and chemokines (IL-1β, -2, -4, -10, MIP-3α, or MCP-1, all from R&D Systems, Wiesbaden, Germany), and with the mitogen ConA (1 μg/ml, Sigma-Aldrich). After stimulation, cells were collected on ice, centrifuged, and pellets were stored at −80°C.

Cells were pretreated with permeable small molecule inhibitors (Calbiochem, EMD Chemicals Inc., an Affiliate of Merck KgaA, Darmstadt, Germany) prior to the addition of the stimulants. Pyridon 6, a pan-JAK inhibitor, was applied at concentrations of 0.16, 0.3, 0.6 and 1 μM for 30 min [53]. To inhibit JAKs 1 and 3, the active metabolite of leflunomide, A771726, was added for 120 min (75 and 125 μM) [54]. The STAT6 inhibitor cyclic pifithrin-α was applied for 30 min (0.1, 0.5, 1, 5, 10 and 50 μM) [55].

### Cell transfection and decoy oligonucleotide-experiments

Double-stranded decoy oligonucleotides (TIBMOLBIOL, Berlin, Germany) providing binding motifs for STAT6 (5^′^-gATCCTACTTCATggAAgAAT-3^′^), STAT1/3 (5^′^- gATCgAgTTTACgAgAACTC-3^′^), or STAT5 (5^′^-gATCgCATTTCggAgAAgACg-3^′^) were prepared as described without chemical modifications [56]. Cells were diluted in antibiotic-free culture medium containing 10% serum and were plated for transfection on 6-well-plates (10 cm^2^ surface area) using the BLOCKiT^TM^ Transfection Kit (Invitrogen GmbH, Karlsruhe, Germany) according to the manufacturer’s instructions. The transfection reagent Lipofectamine 2000^TM^ (5 μl/well) was mixed with 50, 100 or 200 pmol decoy oligonucleotide solutions to allow complex formation prior to addition of the mixture to the cells. Cells were then incubated at 37°C and 5% CO_2_. A non-target, fluorescein-labeled double-stranded RNA oligomer (BLOCKiT^TM^ fluorescent oligo) was used as an indicator of transfection efficiency. Uptake of the BLOCKiT oligo was observed already at 6 h post transfection and persisted for at least 24 h in the cells, as assessed using a fluorescence microscope. Accordingly, medium was replaced 24 h after transfection by pure RPMI-1640 medium, then cytokine was added and cells were incubated for another 2 h at 37°C and 5% CO_2_. Thereafter, cells were collected on ice, centrifuged, and pellets were stored at −80°C until POMC exon 2–3 mRNA was assayed using qRT-PCR.

### Radioimmunoassay and Enzyme Immuno Assay

Cellular content of beta-endorphin was determined by measuring immunoreactive beta-endorphin in cell lysates using a rat radioimmunoassay (RIA) kit according to the manufacturer’s instructions (Phoenix Peptides Inc., Burlingame, CA, USA) and as previously described [21,57]. Briefly, lymphocytes were lyzed by sonication (15 sec, 1 impulse/sec) at a concentration of approximately 3 × 10^6^ cells per 100 μl assay buffer and beta-endorphin immunoreactivity was determined in 100 μl of the lysates in duplicate.

The release of beta-endorphin was determined in cell supernatants using a human/rat-fluorescent EIA kit according to the manufacturer’s instructions (Phoenix Peptides Inc.) as previosly described [7] Briefly, release was induced by incubation of approximately 6× 10^6^ cells/120 μl RPMI-1640 medium containing 10 μM ionomycin (Sigma-Aldrich). Cells were then incubated for 7 min at 37°C and 600 rpm in a thermal heating block, chilled on ice, and centrifuged for 10 min at 450 × g and 4°C. Wells of EIA plates were loaded with 50 μl of the supernatants each; beta-endorphin immunoreactivity was assessed in duplicate.

### Western Blot analysis

Western Blotting was performed as previously described [46]. Briefly, cells were sonicated and homogenized in RIPA buffer [50 mM Tris–HCl (pH 8.0); 150 mM NaCl; 1% Nonidet P-40 (v/v); 0.5% Desoxycholat (w/v); 0.1% SDS (w/v)] in the presence of protease and phosphatase inhibitors (Complete mini and PhosSTOP tablets, Roche). Proteins (30–50 μg/sample) were subjected to polyacrylamide gel-electrophoresis, the gels were composed of an upper stacking and a lower resolving part according to the method of Laemmli [58]. After separation, proteins were transferred at 350 mA/60 min to Immobilon-P membranes (Millipore Corperation, Billerica, MA, USA). Membranes were blocked in Tris buffered saline containing 2.5% bovine serum albumin and 0.1% Tween-20 for at least 30 min at room temperature. After blocking, blots were *sequentially* probed with the following polyclonal rabbit antibodies overnight at 4°C: anti-phospho-STAT1 (Tyrosine 701, 84/91 kDa), anti-STAT1 (84/91 kDa), anti-phospho-STAT3 (Tyrosine 705, 79/86 kDa), anti-phospho-STAT3 (Serine 727, 86 kDa), anti-acetyl-STAT3 (Lysine 685, 79/86 kDa), anti-STAT3 (79/86 kDa), anti-phospho-STAT5 (Tyrosine 694, 90 kDa), anti-STAT5 (90 kDa), anti-phospho-STAT6 (Tyrosine 641, 110 kDa), anti-STAT6 (110 kDa), anti-phospho-Akt (Threonine 308, 60 kDa), anti-phospho-Akt (Serine 473, 60 kDa), anti-Akt (60 kDa), anti-phospho-p44/42 (recognizes p42 when phosphorylated at Threonine 183/Tyrosine 185 in the rat, 42 kDa), anti-p44/42 (42/44 kDa), as well as with anti-phospho-p38 (Threonine 180/Tyrosine 182, 38 kDA) and anti-p38 (38 kDA). All antibodies were diluted 1/1000 in blocking buffer and purchased from Cell Signaling Technologies (Danvers, Massachusetts, USA). After incubation with peroxidase-conjugated secondary antibodies (goat anti-rabbit IgG and rabbit anti-mouse IgG purchased from Jackson ImmunoResearch Europe Ltd., Suffolk, UK) diluted 1/5000 in blocking buffer, immunoreactive bands were visualized using an ECL system. Peroxidase-conjugated anti-beta-actin (42 kDa) was purchases from Sigma-Aldrich and diluted 1:50,000 in blocking buffer; this antibody renders the use of a secondary antibody before overlay of the blot with ECL solution unnecessary. Exposure time of the blots to autoradiograph hyperfilms was 10 to 120 s. Bound antibodies were removed by stripping for 15 min at 50°C in 62.5 mM Tris–HCl containing 100 mM beta-Mercaptoethanol and 2% SDS. Controls included reprobing upon omission of primary or secondary antibodies.

### PCR primers and quantitative real-time PCR (qRT-PCR)

Primers were designed to amplify POMC and ribosomal protein L19 mRNA transcripts using OLIGO Primer Analysis Software Version 5.0 for Windows. Oligodeoxynucleotides were synthesized and purified by TIBMOLBIOL. Real-time PCR assays were performed using the Fast start DNA Master SYBR Green I assay (Roche) according to the instructions of the manufacturer in a LightCycler 1.5 instrument including melting curve analyses. Positive controls contained pituitary cDNA; negative controls contained double-distilled H_2_O or RT^-^ cDNA. Amplification was performed as detailed in Sitte et al. [21], all samples but positive and negative controls were run in duplicate. For some measurements, sensitivity for POMC mRNA amplification was enhanced using a semi-nested real-time PCR protocol as previously described [21]. The amount of POMC exon 2–3 transcripts (sense primer: 5^′^-CCCTCCTGCTTCAGACCTCCA-3^′^, antisense primer: 5^′^-TCTCTTCCTCCGCACGCCTCT-3^′^) was normalized to the expression levels of ribosomal protein L19 using exon 2–5 spanning primers (sense primer: 5^′^-AATCGCCAATGCCAACTCTCG-3^′^, antisense primer: 5^′^-TGCTCCATGAGAATCCGCTTG-3^′^), treatment effects were evaluated by applying the delta-delta CP method as detailed below.

### Immunosuppression of rats and measurement of nociceptive thresholds

Rats were handled daily for 4 days. They were treated thrice at intervals of 48 h intraperitoneally (i.p.) with cyclophosphamide (CTX, Baxter Oncology) to induce depletion of immune cells as previously described [49]; a single i.pl. CFA injection into the right hind paw was given 72 h after the first CTX-injection. At 96 h post CFA-inoculation, immunosuppressed rats received purified T lymphocytes into inflamed paws (i.pl.); control animals were injected with vehicle (PBS). These T cells were obtained from pooled axilliary and inguinal lymph nodes of healthy donor rats as detailed above. Cells were treated for 24 h with/without ConA (1 μg/ml), IL-4 (10 ng/ml), or ConA plus IL-4 ex vivo. Then cell suspensions were depleted of MHC class II receptor^+^ and CD45RA^+^ cells (dendritic cells, monocytes/macrophages, and B lymphocytes) using magnetic cell sorting columns, anti-rat MHC class II receptor and anti-rat CD45RA beads (Miltenyi Biotec, Bergisch Gladbach, Germany), similar to Sitte et al. 2007 [21]. This procedure revealed > 95% pure T cell suspensions that were reconstituted at 1× 10^5^, 5× 10^5^ and 10× 10^5^ cells per 50 μl PBS for i.pl. injections. In the first set of experiments, animals received i.pl. CRF (1.5 ng/50 μl) to induce opioid peptide release 10 min after i.pl. T cell administration. In the second experiment, naloxone-methiodide (NLX; 10 mg/kg) or vehicle (saline) were injected subcutaneously (s.c.) 5 min after i.pl. T cell administration. Another 5 min later the animals received i.pl. CRF (1.5 ng/50 μl). Another group of immunocompetent rats received s.c. NLX or vehicle, followed by i.pl. CRF.

Mechanical hyperalgesia was tested by measuring paw pressure thresholds (PPT) (modified Randall–Selitto test; Ugo Basile) as previously described [59]. Measurements were performed immediately before (baseline) and 7 min after T cell transfer, as well as 5 min post CRF injection. Three consecutive trials, separated by 10 s intervals each, were conducted and the average was calculated. The sequence of left and right paws was alternated between animals to avoid bias. The experimenter was blind to the treatment.

### Data processing

POMC and ribosomal protein L19 qRT-PCR data were analyzed using the LightCycler software 3.5 (Roche). Levels of transcripts were assessed as crossing points (CP) when specific amplification exceeded background fluorescence using the Second Derivative Maximum analysis method of the system. Average PCR efficiencies were 1.89 for rpL19 and 1.82 for POMC exon 2–3 transcripts. All data were subsequently extrapolated using MS Excel 2003. Differences between treated and untreated cells are shown as mean POMC exon 2–3 mRNA ratios ± SEM and were calculated by applying the delta-delta method: Ratio = (PCR efficiency of POMC)^delta CP_POMC(control-sample)_/(PCR efficiency of rpL19)^delta CP_rpL19(control-sample)_. Based on this equation, enhanced POMC mRNA expression gives ratios > 1, decreased POMC mRNA expression gives ratios < 1. The cytokine array and western blot hyperfilms were scanned at 400 dpi and inverted for analysis by optical densitometry using Image J software 1.37v (Wayne Rasband, National Institute of Mental Health, Bethesda, Maryland, USA). Data are presented as mean % expression of loading control ± SEM after background correction.

### Statistical analysis

All data were analyzed with GraphPad Prism Version 4.01 for Windows (GraphPad Software, USA). Normalized cytokine array data were analyzed using unpaired *t*-test with Welch’s correction to account for the number of experiments performed. Statistical analysis was performed on normalized CP values (e.g. CP_POMC_-CP_rpL19)_ in case of the qRT-PCR data; beta-endorphin immunoreactivity values (cellular content as well as amount in the supernatant in pg) were normalized to one million cells. Statistical significance with respect to qRT-PCR data was calculated using the non-parametric Wilcoxon signed rank test if two groups were compared. Multiple comparisons of matched qRT-PCR, RIA, EIA, and Western Blot data were performed using the non-parametric Friedman Test; post-hoc comparisons were performed by Dunn’s test. Behavioral data (average PPT values) were analyzed by Two-Way repeated measures (RM) ANOVA and Bonferroni correction for multiple comparisons. For all tests, statistical significance was considered if P < 0.05.

## Abbreviations

Akt: Proteine kinase B; ConA: Concanavalin A; CFA: Complete Freund’s Adjuvant; CP: Crossing point; CRF: Corticotropin releasing factor; ERK: Extracellular-signal regulated kinases; JAK: Janus kinase; POMC: Proopiomelanocortin; qRT-PCR: Quantitative real-time-polymerase chain reaction; RIA: Radioimmunoassay; RT: Reverse transcriptase; STAT: Signal transducer and activator of transcription.

## Competing interests

The authors declare that they have no competing interests.

## Authors’ contributions

Melanie Busch-Dienstfertig conceived the study, established in vitro experiments, analyzed data, and wrote the paper; Dominika Labuz performed behavioral in vivo experiments and analyzed data; Theresa Wolfram performed cell stimulation, PCR experiments and analyzed data, Nicole Vogel performed and optimized Western Blotting and cell stimulation experiments and analyzed data; Christoph Stein participated in the study design and coordination and drafted the manuscript. All authors read and approved the final manuscript.
